
JOURNAL INFORMATION
==============================
NLM Title Abbreviation: ScientificWorldJournal
Journal ID: ScientificWorldJournal
NLM Journal ID: 101131163
Journal ID: TSWJ

The Scientific World Journal

ISSN: 2356-6140
EISSN: 1537-744X
Publisher: Wiley

ARTICLE INFORMATION
==============================
PMCID: PMC5974876
PMID: 12858011
DOI: 10.1100/tsw.2003.47
Article ID: 575970
Article version: 1
Subjects: Directions in Science

Oscillatory Behavior of the Nuclear Localization of the Transcription Factors Msn2 and Msn4 in Response to Stress in Yeast

Jacquet Michel *
Renault Georges
Lallet Sylvie
De Mey Jan
Goldbeter Albert
Laboratoire Information Génétique et Développement, Institut de Gén#233;tique et Microbiologie, CNRS/UPS UMR 8621, Bldg 400, Universit#233; Paris-Sud Orsay, F-914905 Orsay cedex, France
*Michel Jacquet: michel.jacquet@igmors.u-psud.frAcademic Editor: Martin Goette

Publication date: 2003
Electronic publication date: 2003 Jul 2
Volume: 3
First page: 609
Last page: 612
Copyright: Copyright © 2003 Michel Jacquet et al.
Copyright year: 2003
License: This is an open access article distributed under the Creative Commons Attribution License, which permits unrestricted use, distribution, and reproduction in any medium, provided the original work is properly cited.
License URL: https://creativecommons.org/licenses/by/3.0/

Keywords: transcriptional regulation, stress, nuclear import and export, computational biology, cellular imaging, oscillations, Saccharomyces cerevisiae

Funding: Centre national de la recherche scientifique University of Paris-Sud

==============================
**Directions in Science**
*TheScientificWorldJOURNAL* (2003) 3, 609–612
ISSN 1537-744X; DOI 10.1100/tsw.2003.47

### Oscillatory Behavior of the Nuclear Localization of the Transcription Factors Msn2 and Msn4 in Response to Stress in Yeast

Michel Jacquet[1,*], Georges Renault[1], Sylvie Lallet[1], Jan De Mey[2], Albert Goldbeter[3]

*[1]Laboratoire Information Génétique et Développement, Institut de Génétique et Microbiologie, CNRS/UPS UMR 8621, Bldg 400, Université Paris-Sud Orsay, F-914905 Orsay cedex, France; [2]Institut Curie - CNRS, UMR 146, F-91405 Orsay cedex, France (Present address: École Supérieure de Biotechnologie de Strasbourg, C.N.R.S. - U.M.R. 7100, 1, Bld Sébastien Brant, F-67400 Illkirch-Graffenstaden, France); [3]Unité de Chronobiologie théorique, Faculté des Sciences, Université Libre de Bruxelles, C.P. 231, B-1050 Brussels, Belgium*

E-mails: michel.jacquet@igmors.u-psud.fr; renault@igmors.u-psud.fr; lallet@igmors.u-psud.fr; jan.demey@esbs.u-strasbg.fr.; agoldbet@pop.ulb.ac.be

*Received May 19, 2003; Accepted June 16, 2003; Published July 2, 2003*

---

**KEY WORDS:** transcriptional regulation, stress, nuclear import and export, computational biology, cellular imaging, oscillations, *Saccharomyces cerevisiae*

**DOMAINS:** cell biology, intracellular signaling, microscopy, computational biology, modeling, fungal biology, molecular biology, biochemistry

---

Rhythmic behaviors of molecular components of the cell represent a fascinating aspect of biology because they imply complex feedback regulations[1,2]. Biological oscillatory phenomena have been observed over a very large range of time periods, extending from fractions of a second to years. The involvement of oscillations in gene expression has been characterized more recently, with the finding that circadian rhythms rely upon negative autoregulatory feedback giving rise to periodic gene expression[3]. Oscillations that involve the negative regulation of transcription have also been described for P53[4], NFκB[5], and somite formation during development[6].

In a recent study published in *Journal of Cell Biology*, we described a new class of oscillatory behavior for transcriptional regulators, namely, the rapid nucleocytoplasmic shuttling of two transactivators of the yeast *Saccharomyces cerevisiae*, Msn2 and Msn4, which mediate a general stress response.

In *Saccharomyces cerevisiae*, a large set of genes is induced either by nutritional limitations or upon various environmental stresses such as heat, oxidative, osmotic or even ethanol shock[7 ,8 ,9]. Such an induction complements the more specific responses elicited by the

*Corresponding author.
©2003 with author.

different environmental changes[10]. This phenomenon, described as the general stress response, is generally transient, with an intensity and duration that depend upon the strength of the stress[8]. The two related transcriptional activators Msn2 and Msn4 mediate these effects through stress response elements in the promoters of regulated genes[11,12]. In response to stress, Msn2 and Msn4 migrate from the cytoplasm to the nucleus[13]. Both of them contain a nuclear localization signal (NLS) and possibly also nuclear export signal(s) (NES)[14]. The cAMP-PKA signaling pathway, which is functional during growth, controls negatively Msn2 and Msn4[7,15 ] and prevents their nuclear localization by acting both upon import and export[14]. How the different stress signals are transmitted to these factors is not yet fully elucidated. Nevertheless, it is known that their activation correlates with hyperphosphorylation[16]. The NLS appears to be negatively controlled by the PKA, but other protein kinases are also involved, since in the absence of PKA, Msn2 and Msn4 are hyperphosphorylated and fully active in transcription.

While searching for determination of the kinetic parameters of nuclear migration in response to stress we found, using time-lapse microscopy and and Msn2 fused to the green fluorescent protein (GFP) [13], that such a migration was induced even without applying external stress. Migration to the nucleus was prevented when using much weaker illumination. Thus, the light emitted by the fluorescence microscope was sufficient to be sensed as a stress by the cell. This first result, of particular importance to cell biologists, indicates that the microscopic observation of GFP-containing cells might induce stress-related modifications within the observed cells. The second and more surprising observation was the repetitive shuttling of the overall Msn2-GFP molecular population back and forth between cytoplasm and nucleus. The periodicity of this oscillatory nucleocytoplasmic shuttling was in the range of 4 to 6 min and differed from cell to cell. This behavior was also induced by different stresses such as oxidative, osmotic, and ethanol shock.

Such a rapid oscillatory shuttling is hardly compatible with a control at the level of expression of a regulatory element, as found for periodic transcription mediated by PER (period) and TIM (timeless) proteinsin circadian rhythms [17], P53 and mdm2[4], or NFκB and its inhibitor[5]. Indeed, we showed that the oscillatory nucleocytoplasmic shuttling continues when cycloheximide, an inhibitor of protein synthesis, is added to the cells, and even when the DNA binding domain of Msn2 is removed. Therefore, this phenomenon inaugurates a new class of oscillatory mechanism for a transcriptional activator. Among the putative effectors, the role of the cAMP-PKA pathway was investigated. As previously described, high cAMP levels in a phosphodiesterase mutant prevent nuclear migration, while the absence of the catalytic subunits of PKA results in permanently sending Msn2-GFP in the nucleus. Interestingly, Msn4-GFP, which also oscillates in response to light and stress, was still oscillating in a mutant defective of PKA, thus showing either a differential sensitivity to cAMP and stress or a different control for nuclear localization.

The cyclical switching between a cytoplasmic and a nuclear localization of Msn2 can be explained either by the coupling to an external oscillatory process of its transport into and out of the nucleus, or by an autoregulatory loop controlling the subcellular localization of the transcription factor. A plausible model for an autoregulatory loop is proposed in this paper. To provide a delay which could strengthen the nonlinearity required to produce oscillations in the cycling between cytoplasm and nucleus we propose the existence of a feed-forward loop composed of a double switch. This switch could be composed of protein kinases and phosphatases acting in a phosphorylation-dephosphorylation cascade that would promote the resetting of the transactivator cycle. Such a putative model can account for the observations and provides a framework for comprehending the conditions of occurrence of the periodic phenomenon. In particular the model predicts that oscillations should occur only within a given range of stress and cellular sensitivity.

The description of this unpredicted behavior of transcriptional activators highlights a novel facet of transcriptional regulation. By a rapid shuttling between cytoplasm and nucleus the transactivators could sense the state of the cell and permit a fast adaptation to environmental stimuli. It shows that the dynamics of molecular processes can be integrated in a more complex fashion than previously thought. It also provides an example for the emergence of integrated functions in the cell which cannot simply be predicted by the properties of the individual components. The results emphasize the interest of developing *in vivo* time-resolved imaging of cellular components. Finally, this study illustrates how computational cell biology can be used to throw light on the origin of complex dynamic behavior at the cellular level. Along the experimental approach, models and numerical simulations are playing an increasingly important role in molecular and cell biology.

### ACKNOWLEDGEMENTS

This work was supported by the CNRS, the University of Paris-Sud, ACI “interface physique-chimie-biologie: dynamique et réactivité des assemblages biologiques” and ARC grant no. 5693 to MJ, ARC grant no. 9725 to JDM, and grant no. 3.4607.99 from the F.R.S.M. (Belgium) to AG.

This paper was excerpted from Jacquet, M. et al. (2003) Oscillatory nucleocytoplasmic shuttling of the general stress response transcriptional activators Msn2 and Msn4 in *Saccharomyces cerevisiae*. *J. Cell Biol.* **161**, 497–505.

### REFERENCES

1. Goldbeter, A. (1996) *Biochemical Oscillations and Cellular Rhythms*. Cambridge University Press, Cambridge, UK.
2. Goldbeter, A. (2002) Computational approaches to cellular rhythms. *Nature* **420**, 238–245.
3. Sehgal, A., Rothenfluh-Hilfiker, A., Hunter-Ensor, M., Chen, Y., Myers, M.P., and Young, M.W. (1995) Rhythmic expression of timeless: a basis for promoting circadian cycles in period gene autoregulation. *Science* **270**, 808–810.
4. Lev Bar-Or, R., Maya, R., Segel, L.A., Alon, U., Levine, A.J., and Oren, M., (2000) Generation of oscillations by the p53-Mdm2 feedback loop: a theoretical and experimental study. *Proc. Natl. Acad. Sci. U.S.A.* **97**, 11250–11255.
5. Hoffmann, A., Levchenko, A., Scott, M.L. and Baltimore, D. (2002) The IkappaB-NF-kappaB signaling module: temporal control and selective gene activation. *Science* **298**, 1241–1245.
6. Dale, J.K., Maroto, M., Dequeant, M.L., Malapert, P., McGrew, M., and Pourquie, O. (2003) Periodic Notch inhibition by Lunatic Fringe underlies the chick segmentation clock. *Nature* **421**, 275–278.
7. Boy-Marcotte, E., Perrot, M., Bussereau, F., Boucherie, H., and Jacquet, M. (1998) Msn2p and Msn4p control a large number of genes induced at the diauxic transition which are repressed by cyclic AMP in *Saccharomyces cerevisiae*. *J. Bacteriol.* **180**, 1044–1052.
8. Gasch, A.P., Spellman, P.T., Kao, C.M., Carmel-Harel, O., Eisen, M.B., Storz, G., Botstein, D., and Brown, P.O. (2000) Genomic expression programs in the response of yeast cells to environmental changes. *Mol. Biol. Cell.* **11**, 4241–4257.
9. Causton, H.C., Ren, B., Koh, S.S., Harbison, C.T., Kanin, E., Jennings, E.G., Lee, T.I., True, H.L., Lander, E.S., and Young, R.A. (2001) Remodeling of yeast genome expression in response to environmental changes. *Mol. Biol. Cell.* **12**, 323–337.
10. Boy-Marcotte, E., Lagniel, G., Perrot, M., Bussereau, F., Boudsocq, A., Jacquet, M., and Labarre, J. (1999) The heat shock response in yeast: differential regulations and contributions of the Msn2p/Msn4p and Hsf1p regulons. *Mol. Microbiol.* **33**, 274–283.
11. Martinez-Pastor, M.T., Marchler, G., Schuller, C., Marchler-Bauer, A., Ruis, H., and Estruch, F. (1996) The *Saccharomyces cerevisiae* zinc finger proteins Msn2p and Msn4p are required for transcriptional induction through the stress response element (STRE). *Embo J.* **15**, 2227–2235.
12. Schmitt, A.P. and McEntee, K. (1996) Msn2p, a zinc finger DNA-binding protein, is the transcriptional activator of the multistress response in *Saccharomyces cerevisiae*. *Proc. Natl. Acad. Sci. U.S.A.* **93**, 5777–5782.
13. Gorner, W., Durchschlag, E., Martinez-Pastor, M.T., Estruch, F., Ammerer, G., Hamilton, B., Ruis, H., and Schuller, C. (1998) Nuclear localization of the C2H2 zinc finger protein Msn2p is regulated by stress and protein kinase A activity. *Genes Dev.* **12**, 586–597.

14. Gorner, W., Durchschlag, E., Wolf, J., Brown, E.L., Ammerer, G., Ruis, H., and Schuller, C. (2002) Acute glucose starvation activates the nuclear localization signal of a stress-specific yeast transcription factor. *Embo J.* **21**, 135–144.
15. Smith, A., Ward, M.P., and Garrett, S. (1998) Yeast PKA represses Msn2p/Msn4p-dependent gene expression to regulate growth, stress response and glycogen accumulation. *Embo J.* **17**, 3556–3564.
16. Garreau, H., Hasan, R.N., Renault, G., Estruch, F., Boy-Marcotte, E., and Jacquet, M. (2000) Hyperphosphorylation of Msn2p and Msn4p in response to heat shock and the diauxic shift is inhibited by cAMP in *Saccharomyces cerevisiae*. *Microbiology* **146**, 2113–2120.
17. Darlington, T.K., Wager-Smith, K., Ceriani, M.F., Staknis, D., Gekakis, N., Steeves, T.D., Weitz, C.J., Takahashi, J.S., and Kay, S.A. (1998) Closing the circadian loop: CLOCK-induced transcription of its own inhibitors per and tim. *Science* **280**, 1599–1603.

**This article should be referenced as follows:**

Jacquet, M., Renault, G., Lallet, S., De May, J., and Goldbeter, A. (2003) Oscillatory behavior of the nuclear localization of the transcription factors Msn2 and Msn4 in response to stress in yeast. *TheScientificWorldJOURNAL* **3**, 609–612.

**Handling Editor:**

Martin Gotte, Editorial Board Member for *Biochemistry* and Principal Editor for *Cell Biology* — domains of *TheScientificWorldJOURNAL*.

### BIOSKETCH

Michel Jacquet is Professor at the University Paris-Sud and has been responsible for the PHD program “Molecular Biology of the Cell” since 1990. He is director of the “Institute of Genetic and Microbiology” within the University in Orsay since 2001. He was president of the Department of Biology of the University between 1998 and 2000 and was president of the French Society for Cell Biology from 1997 to 1999. His research interests are Molecular and cell biology, cellular signaling, gene regulation, signal transduction, and computational biology.

He main interest are cellular regulation at the level of gene expression and in signalling pathways. He has been working on various biological organisms, such as, *E. coli*, retroviruses, cell lines, *Dictyostelium* and, for the past 18 years, *Saccharomyces cerevisiae*. He was also involved in the sequencing of the yeast genome from 1990 to 1996.
